# Patient–Physician Differences in Desired Characteristics of NSAID Plasters: An Online Survey

**DOI:** 10.1155/2017/5787854

**Published:** 2017-11-26

**Authors:** On Takeda, Daisuke Chiba, Yasuyuki Ishibashi, Eiichi Tsuda

**Affiliations:** ^1^Department of Orthopaedic Surgery, Hirosaki University Graduate School of Medicine, Hirosaki, Japan; ^2^Department of Rehabilitation Medicine, Hirosaki University Graduate School of Medicine, Hirosaki, Japan

## Abstract

In Japan, adhesive skin plasters containing nonsteroidal anti-inflammatory drugs (NSAIDs) are frequently used to treat pain of musculoskeletal origin. There are many reports on their efficacy but few on patients' impressions of usability or levels of satisfaction. *Objectives*. To elucidate the differences in perception between patients and physicians concerning NSAID plasters. *Subjects*. We conducted two surveys about NSAID plasters on patients and physicians. 600 patients currently using plasters and 200 physicians currently prescribing NSAID plasters were studied. *Methods*. Questionnaire included items concerning usage, efficacy and side effects, site and intensity of their pain, pain management strategies, characteristics they desired from NSAID plasters, and their satisfaction with them. *Results*. The characteristic most frequently reported as desirable by patients was analgesic efficacy, followed by avoiding skin irritation and low medication cost. The characteristics most frequently reported as desirable by physicians were analgesic efficacy, alignment with patient preference, safety to skin, and comfort when applied. Our survey revealed that both patients and physicians prioritized analgesic efficacy of NSAID plasters. However, approximately half of the patients and physicians were unsatisfied with the analgesic efficacy of plasters. *Conclusions*. Physicians may improve patient satisfaction by discussing analgesic efficacy, skin complications, and price with patients before prescription.

## 1. Introduction

Topical plasters containing nonsteroidal anti-inflammatory drugs (NSAIDs) are designed to relieve pain of musculoskeletal origin and are indicated for a wide variety of diseases. They are frequently used as a route of administration of analgesics in Japan. Causing gastrointestinal complications less frequently than oral analgesics, NSAID plasters are safer for elderly patients [1, 2] and will likely be used more frequently as society ages. Many previous studies have reported on the pharmacological strengths of NSAID plasters, which include analgesic efficacy and reduced side effects [3, 4]. However, few studies have considered which aspects of these plasters concern the patients who actually use them, for example, usability, treatment expectations, and satisfaction [5]. It has been reported that only 19% of people suffering from chronic musculoskeletal pain seek medical care, compared to 20% who seek alternative therapies such as massage and acupuncture [6]. Such results are suggestive of a lack of efficacy or satisfaction from medical treatment in these conditions.

Aiming to examine factors that could potentially raise patient satisfaction with NSAID plasters, we conducted two online surveys that enquired with patients and physicians about their treatment expectations of, and satisfaction with, such medications and compared their responses.

## 2. Subjects and Methods

### 2.1. Patient Survey

Our patient survey was an online survey run for six days (March 25–30, 2015), administered using an existing mailing list of people in the general population. Of the 12,083 respondents, those who were not currently using NSAID plasters were excluded. The eligible respondents were stratified by age (20–44, 45–59, and 60–89 y) and sex. The first 100 were included in each respective age group of both sexes for a total of 600 (average age = 53.2 y). After 100 respondents were included for each category, further responses were excluded. Questionnaire items asked patients about the department or clinic that prescribed their NSAID plasters, the site and intensity of their pain, their pain management strategy, expectations and satisfaction of treatment with NSAID plasters, and their mental associations with the expression “comfort” when applied (multiple responses allowed).

### 2.2. Physician Survey

Our physician survey was an online survey run for three days (March 25–27, 2015), administered using an existing mailing list of physicians. Of the 995 respondents, those who prescribed NSAID plasters to more than 50 patients in the past month were included. The respondents were sorted by specialty and practice type (outpatient clinic or hospital). 50 respondents who specialized in orthopedics and general practice (the specialties that most often treat musculoskeletal pain) for each practice type were chosen in chronological order for a total of 200 physicians. After 50 respondents were included for each category, further responses were excluded. Questionnaire items asked physicians about their affiliated clinical department, their concerns and priorities when prescribing NSAID plasters, their satisfaction with existing drugs, their estimations of patient satisfaction, and specific factors that they associate with the expression “comfort” when applied.

The types and available formulations of the plasters included in this survey are shown in Table 1. The specific dose and location of application were not included in the questionnaire.

This study was approved by the Hirosaki University Graduate School of Medicine Ethics Committee.

## 3. Results

### 3.1. Patient Survey

223 (37.2%) patients used NSAID plasters in combination with oral analgesics. The prescribing entity was most commonly a physician from an orthopedic surgery department (67%), followed by general internal medicine department (17%), general surgery department (3%), and other departments (1% each). In terms of frequency of use, 31% of patients used NSAID plasters almost every day, while 35% used them 1–3 times per week.

Patients most frequently reported pain in their lower back (68.3%), followed by the shoulder (58.0%), the knee (38.2%), and the neck (37.8%). 74.7% of patients complained of pain at multiple sites (Table 2). Women had a higher incidence of shoulder and knee pain. Incidence of knee pain increased with each rise in age group, while neck pain decreased. Differences in the pain management strategy due to the pain site were observed, with patients visiting medical institutions most frequently for lower back pain (82.9%) and least frequently for hip-joint pain (54.1%) (Table 3).

**Figure 1:**
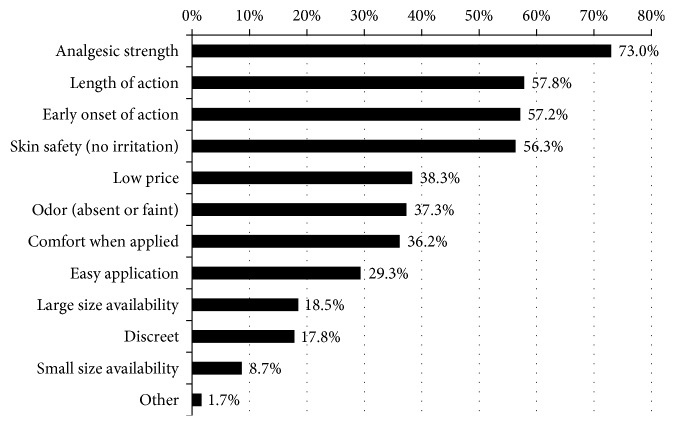
Patient survey: desired characteristics of plasters (multiple responses allowed). Patients prioritized analgesic efficacy and other items related to efficacy. Safety and cost were also important.

Items related to analgesic efficacy were highly ranked among patients' desired characteristics of NSAID plasters, including strong pain relief (73.0%), length of action (57.8%), and early onset of action (57.2%). The next most desired characteristics were avoiding skin irritation (56.3%) and low price (38.3%) (Figure 1). Young adult patients prioritized medication price higher than older patients; all other desirable aspects were more frequently cited by older patients.

**Figure 2:**
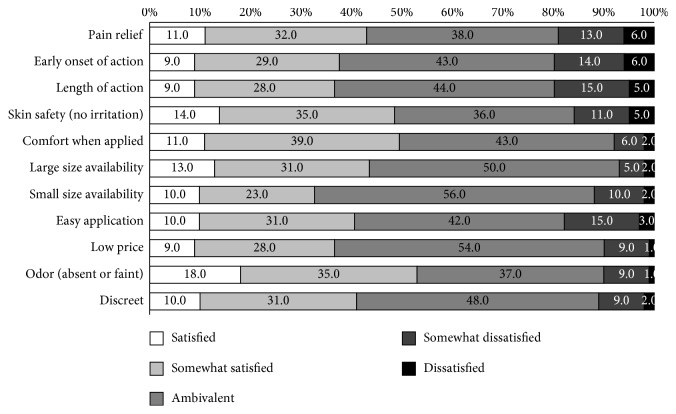
Patient survey: item-by-item satisfaction with NSAID plasters. Low satisfaction was reported in factors related to efficacy, cost, and availability of small size. High satisfaction was reported with skin safety, comfort when applied, and odor.

Patients' perceptions of NSAID plasters' analgesic effects differed by the affected site. Although 20–33% responded that they did not feel any noticeable improvement, the majority of patients reported improvement of symptoms (Table 4). With regard to NSAID plasters' analgesic effects, 43.0% of patients responded that they were either satisfied or somewhat satisfied, while the remaining 57.0% were either ambivalent or unsatisfied. Patients also reported that they were satisfied with plasters' characteristics in terms of avoiding skin irritation (49.0%), comfort when applied (50.0%), and price (37.0%) (Figure 2).

### 3.2. Physician Survey

**Figure 3:**
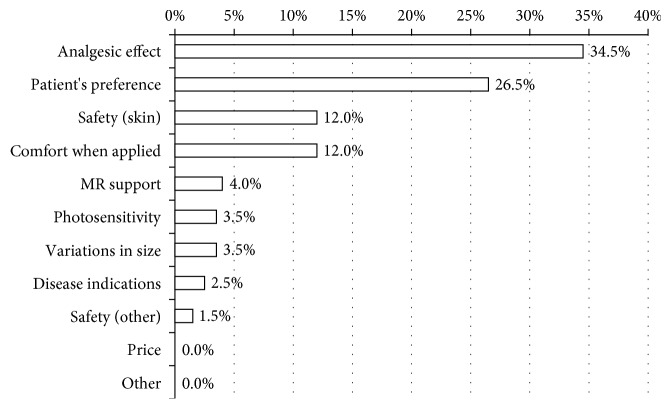
Physician survey: single most desired characteristic of plasters (%, single response, and *n* = 200). Analgesic effect and patient's preference were the most frequent responses. Cost and systemic adverse effects were lowest in priority. MR: medical representative.

**Figure 4:**
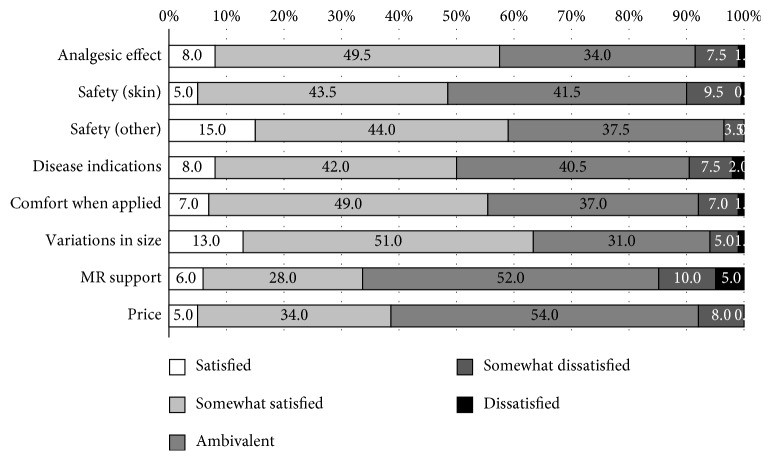
Physician survey: item-by-item satisfaction with NSAID plasters. Satisfaction of estimated analgesic effect, variation in size, and systemic safety were high. Satisfaction of MR support and price was low, but the majority of physicians were ambivalent to these factors. MR: medical representative.

In the second survey, 34.5% of physicians reported analgesic effects to be the single most important characteristic desired of NSAID plasters. The next most frequent response was whether the NSAID plaster matched their patient's preference (26.5%), followed by safety to skin (12.0%) and comfort when applied (12.0%) (Figure 3). 57.5% responded that they were satisfied or somewhat satisfied with plasters' analgesic effects. 56.0% were satisfied or somewhat satisfied with comfort when applied, while 39.0% were satisfied with the drugs' prices (Figure 4). Satisfaction with price was low, but the majority of physicians were ambivalent to this factor, reflecting the low priority among physicians.

**Figure 5:**
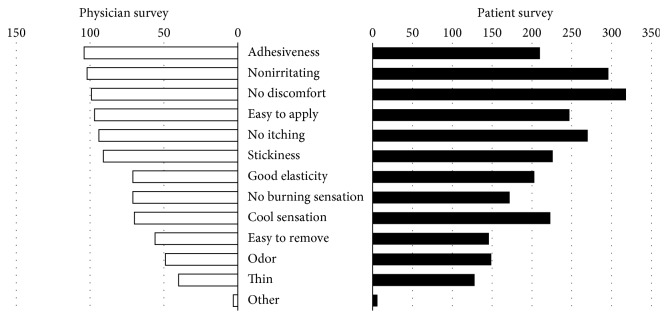
Concepts associated with “comfort” when applied (multiple responses allowed: physician survey (*n* = 200) and patient survey (*n* = 600)). Nonirritating, no discomfort, easy application, and no itching sensation were highly reported among both physicians and patients.

Both patients and physicians were asked about specific factors that they associate with the expression “comfort” when applied. The responses most frequently seen were “not irritating to skin” (patients = 51.0%, physicians = 49.3%), “not itchy” (47.0%, 45.0%), “no discomfort” (49.5%, 53.0%), and “easy to apply” (48.5%, 41.2%) (multiple responses allowed). Physicians also associated the term “comfort” with adhesiveness, but fewer patients felt the same (52.0%, 35.0%, resp.) (Figure 5).

## 4. Discussion

Respondents to our patient survey reported lower-back pain most frequently, followed by pain in the shoulder, knee, and neck, while 74.7% complained of pain at multiple sites. In addition, large differences were observed in rates of visitation to medical institutions depending on the pain site, from a maximum of 82.9% of all patients with lower-back pain to a minimum of 54.1% of all patients with hip-joint pain. This suggests that patients may have different expectations of medical care depending on the site of their pain and may in some cases choose other treatments such as alternative therapies. Patients using NSAID plasters who visited medical institutions comprised a higher proportion of our patient population than in past studies. This is likely because we specifically administered our survey to patients who were prescribed NSAID plasters at a medical institution.

The development and spread of transdermal NSAID plasters have followed a unique path in Japan. They are a frequently prescribed mode of administration for NSAIDs in Japan today, owing to a lower incidence of systemic adverse effects and superior safety of topical administration [2, 3]. The North American and European literature lacks studies on NSAID adhesive plasters, focusing on topical agents instead in the form of gels and creams. Some differences between ointments and plasters exist, but both of these topical agents have been shown to be noninferior in analgesic efficacy to oral analgesics [2, 7–9]. Extensive literature exists on the analgesic efficacy of NSAID topical agents for osteoarthritis of the knee. In their 2014 guidelines, Osteoarthritis Research Society International (OARSI) recommends that patients use topical agents for relief of pain due to knee osteoarthritis, citing superior safety and comparable pain relief to oral analgesics [10].

Our survey revealed that both patients and physicians prioritized analgesic efficacy highest among the desirable characteristics of NSAID plasters. Moving forward, we believe that a detailed examination of the efficacy of NSAID plasters according to specific diseases and disease severity, in combination with the continued development of topical agents with even stronger analgesic efficacy, will help to further improve patient satisfaction.

Both patients and physicians viewed safety to skin as an important characteristic for NSAID plasters. This may reflect the fact that 39.3% of patients have experienced some form of skin complication due to the use of NSAID plaster medications [11]. After analgesic efficacy and skin complications, we found that patients were most concerned with drug prices. Low price was of least concern to physicians among desirable characteristics of NSAID plasters, evidencing a marked difference between the concerns of patients and physicians which may have led to the difference in satisfaction of this aspect. Desire for lower prices was most pronounced in younger patients. This may be due to the Japanese medical system, where younger patients pay 30% of their medication costs, while the elderly pay less according to age. These results suggest that considering medication costs may lead to more patient-orientated prescription practices. Further, development of generic medications will reduce the costs and create greater patient choice, increasing the physician's capability to increase patients' satisfaction concerning drug costs when providing patients with pain management strategies.

Patient–physician differences in satisfaction were also observed for other items. A mere 43.0% of patients were satisfied with NSAID plasters' analgesic efficacy, compared with the estimated satisfaction rate of 57.5% in physicians, suggesting patients may be less satisfied with the treatment than physicians perceive. Failure to meet patients' treatment expectations and functional status have been raised as factors that reduce patient satisfaction in general practice [12]. Skin plasters have been shown to provide comparable analgesic efficacy to oral dosage forms, but patients reported low satisfaction with the former in this study. This may have resulted from individual differences in patients' expectations of treatment efficacy, or in the severity of their preexisting conditions. Also, this study did not include a control group of oral analgesics alone or alternative therapies, so a comparison between modes of therapy was not possible. We believe physicians should fully explain to patients the analgesic efficacy they can expect of NSAID plasters, as well as their limitations, in light of their specific musculoskeletal conditions and severity.

Fewer than half of patients and physicians were satisfied with NSAID plasters in terms of their safety to skin, suggesting there is room for improvement in the design of transdermal plasters. Also, 40% of patients responded that they do not report side effects and preferences to their physician. When considered with the low utilization of medical facilities, side effects and dissatisfaction with plasters are likely to be underrepresented and underrecognized. In light of this, there may be a role for the pharmacist who can educate the patient concerning proper usage and side effects. Pharmacies are more easily accessible and usually have information of medications for multiple medical facilities, making them ideal for identifying and differentiating adverse effects and drug interactions.

Only 20% of physicians considered extradermal side effects, and 1.5% responded that they were the most desired characteristics. Although NSAID plasters are systemically absorbed, systemic side effects are rare. Evans reported that topical NSAIDs did not increase the risk of upper gastrointestinal bleeding in a case-control study [13]. The risk of upper gastrointestinal bleeding due to NSAIDs is said to be dose dependent, suggesting that topical formulations used in excess or absorbed more efficiently may cause harm. In addition, non-dose-dependent side effects should be taken into account, as topical NSAIDs are contraindicated in patients with aspirin-exacerbated asthma and pregnant women. Renal side effects have also been associated with topical NSAID use [14], necessitating physicians to be knowledgeable of systemic absorption.

50.0% of patients were satisfied with the comfort of NSAID plasters. There was a high degree of agreement between patients' and physicians' perception of “comfort” when applied, which may have contributed to a higher satisfaction rate. A factor that was not taken into account was the relationship between adhesiveness and climate. Japan has a very diverse climate, so the temperature and humidity may vary greatly between geographic locations at any given time. Clothing and perspiration may also affect the adhesiveness of plasters and influence satisfaction. This study was not designed to assess differences between locations, but from the available data, there was no difference in responses concerning satisfaction in comfort and the perception of adhesiveness between regions. This may be due to the availability of various sizes, thicknesses, and adhesiveness, which patients may choose for their specific needs. Indeed, 101 (16.8%) of respondents used multiple types of plasters.

Musculoskeletal pain is extremely common: according to the 2013 National Livelihood Survey, respective reported incidences for men and women were 9.22% and 11.82% for lower-back pain, 6.02% and 12.50% for stiff shoulders, and 4.18% and 7.03% for limb-joint pain, respectively. However, a mere 4.22% of men and 5.85% of women visit medical facilities because of lower-back pain.

One study found that only 19% of people visit a medical facility to treat chronic musculoskeletal pain, while 20% use alternative therapies (i.e., massage, physical therapy, and acupuncture) and 55% do not consult with a physician at all [6]. The findings of these previous studies suggest low patient expectation with medical care for the treatment of chronic musculoskeletal pain; the findings of our study corroborate them.

Our investigation had several limitations: as it was an Internet survey, we did not directly examine patients and could not confirm the diagnosis of the underlying diseases. In addition, while 37.3% of patients were taking oral medication in combination with an NSAID plaster, we did not examine the details of their oral medication regimen. Also, as we did not have a control group, we could not compare the satisfaction between different treatment modalities (i.e., plasters, oral medications, and alternative therapies).

## 5. Conclusions

The most desirable characteristics of NSAID plasters according to both patients and physicians were strong analgesic efficacy and avoidance of skin complications. Patients and physicians had relatively high satisfaction with “comfort” of plasters, and their conceptions of the term had much in common. We conclude that when prescribing transdermal NSAID plasters, physicians should be knowledgeable about their analgesic effects, potential skin complications, and prices. Sufficiently explaining these aspects to patients, along with prescribing in consideration of underlying diseases, should accordingly lead to increased satisfaction. “High-absorption” transdermal plasters have been recently developed, which allow for high absorption of NSAIDs into the skin [15, 16]: their increased efficacy may improve patient satisfaction. If they can obviate the need for oral intake of NSAIDs, such plasters may reduce the incidence of adverse gastrointestinal events.

## Figures and Tables

**Table 1:** NSAID types and available doses.

Active NSAID	Base	Dose (mg)	Reported serum concentration, AUC (dose)	Respondents
Flurbiprofen	Lipophilic	20, 40	902 ± 157 ng·hr/ml (40 mg^∗^1)	46
Flurbiprofen	Hydrophilic	40, 80		27

Felbinac	Lipophilic	35, 70	17 ± 2.6 ng·hr/ml (70 mg^∗^4)	33
Felbinac	Hydrophilic	70, 140		26

Diclofenac	Lipophilic	15, 30	1419.4 ± 511.9 ng·hr/ml (30 mg^∗^4)	60

Ketoprofen	Lipophilic	20, 40	2447.83 ± 198.67 ng·hr/ml (20 mg^∗^1)	171
Ketoprofen	Hydrophilic	30, 60		51

Loxoprofen	Lipophilic	50, 100	5.281 ± 1.7 ng·hr/ml (100 mg^∗^1)	150
Loxoprofen	Hydrophilic	100		59

Other	—	—	—	48

Unknown	—	—	—	96

**Table 2:** Patient survey: pain site by gender and age group (multiple responses allowed).

Site of pain	Total	Male	Female	Young (20–44)	Middle-aged (45–59)	Elderly (60–89)
Neck	227 (37.8%)	107 (35.7%)	120 (40.0%)	97 (48.5%)	69 (34.5%)	61 (30.5%)
Shoulder	348 (58.0%)	160 (53.3%)	188 (62.7%)	118 (59.0%)	125 (62.5%)	105 (52.5%)
Arm	127 (21.2%)	57 (19.0%)	70 (23.3%)	47 (23.5%)	45 (22.5%)	35 (17.5%)
Hand/wrist	153 (25.5%)	67 (23.3%)	86 (28.7%)	57 (28.5%)	50 (25.5%)	46 (23.0%)
Upper back	158 (26.3%)	76 (25.3%)	82 (27.3%)	66 (33.0%)	54 (27.0%)	38 (19.0%)
Lower back	410 (68.3%)	207 (69.0%)	203 (67.7%)	146 (73.0%)	124 (62.0%)	140 (70.0%)
Hip (joint)	98 (16.3%)	40 (13.3%)	58 (19.3%)	36 (18.0%)	32 (16.0%)	30 (15.0%)
Knee	229 (38.2%)	100 (33.3%)	129 (43.0%)	61 (30.5%)	70 (35.0%)	98 (49.0%)
Foot/ankle	132 (22.0%)	62 (20.7%)	70 (23.3%)	52 (26.0%)	40 (20.0%)	40 (20.0%)
Other	3 (0.5%)	2 (0.7%)	1 (0.3%)	0 (0.0%)	1 (0.5%)	2 (1.0%)
Total	600	300	300	200	200	200

Incidence of knee pain increased with advancing age group, while neck pain decreased. Women had a higher incidence of shoulder and knee pain.

**Table 3:** Patient survey: pain management strategy by pain site (multiple responses allowed).

Site	Neck	Shoulder	Arm	Hand/wrist	Upper back	Lower back	Hip joint	Knee	Foot/ankle	Other
Total	227	348	127	153	158	410	98	229	132	3
Prescribed medicine (including plasters) at a medical institution (%)	148 (65.2)	242 (69.5)	89 (70.1)	96 (62.7)	89 (56.3)	340 (82.9)	53 (54.1)	159 (69.4)	83 (62.9)	3 (100)
Purchases over-the-counter medicine (including plasters) (%)	58 (25.6)	88 (25.3)	33 (26.0)	36 (23.5)	42 (26.6)	87 (21.2)	17 (17.3)	45 (19.7)	35 (26.5)	—
Receiving massages or rehabilitative therapy (%)	79 (34.8)	98 (28.2)	34 (26.8)	30 (19.6)	46 (29.1)	105 (25.6)	21 (21.4)	36 (15.7)	32 (24.2)	2 (66.7)
Exercise and stretching (%)	79 (34.8)	118 (33.9)	32 (25.2)	30 (19.6)	45 (28.5)	129 (31.5)	36 (36.7)	69 (30.1)	36 (27.3)	—
Other (%)	—	—	—	3 (2.0)	1 (0.6)	3 (0.7)	—	3 (1.3)	1 (0.8)	1 (33.3)
No particular strategy (%)	16 (7.0)	17 (4.9)	8 (6.3)	18 (11.8)	20 (12.7)	13 (3.2)	13 (13.3)	27 (11.8)	10 (7.6)	—

Pain management strategies differed by site. 82.9% of patients with lower-back pain seek medical care while only 54.1% of patients seek medical care for hip-joint pain.

**Table 4:** Patient survey: extent of pain improvement by site (%).

Site	Neck	Shoulder	Arm	Hand/wrist	Upper back	Lower back	Hip joint	Knee	Foot/ankle	Other
Total	148	242	89	96	89	340	53	159	83	3
Pain disappeared almost completely (%)	23 (15.5)	32 (13.2)	12 (13.5)	11 (11.5)	17 (19.1)	51 (15.0)	10 (18.9)	24 (15.1)	14 (16.9)	—
Pain improved, but still slightly remains (%)	84 (56.8)	156 (64.5)	55 (61.8)	66 (68.8)	54 (60.7)	222 (65.3)	32 (60.4)	99 (62.3)	50 (60.2)	2 (66.7)
Pain largely unchanged (%)	41 (27.7)	54 (22.3)	22 (24.7)	19 (19.8)	18 (20.2)	67 (19.7)	11 (20.8)	36 (22.6)	19 (22.9)	1 (33.3)

The majority of patients reported some improvement, while the rates of unchanged patients were high in neck and arm pain.
